# Robust Beamfocusing for Secure NFC with Imperfect CSI

**DOI:** 10.3390/s25041240

**Published:** 2025-02-18

**Authors:** Weijian Chen, Zhiqiang Wei, Zai Yang

**Affiliations:** School of Mathematics and Statistics, Xi’an Jiaotong University, Xi’an 710049, China; chenwj0812@stu.xjtu.edu.cn (W.C.); yangzai@xjtu.edu.cn (Z.Y.)

**Keywords:** artificial noise (AN), beamforming, physical layer security (PLS), multiple-input multiple-output multiple-antenna eavesdropper (MIMOME), near-field communication (NFC)

## Abstract

In this paper, we consider the issue of the physical layer security (PLS) problem between two nodes, i.e., transmitter (Alice) and receiver (Bob), in the presence of an eavesdropper (Eve) in a near-field communication (NFC) system. Notably, massive multiple-input multiple-output (MIMO) arrays significantly increase array aperture, thereby rendering the eavesdroppers more inclined to lurk near the transmission end. This situation necessitates using near-field channel models to more accurately describe channel characteristics. We consider two schemes with imperfect channel estimation information (CSI). The first scheme involves a conventional multiple-input multiple-output multiple-antenna eavesdropper (MIMOME) setup, where Alice simultaneously transmits information signal and artificial noise (AN). In the second scheme, Bob operates in a full-duplex (FD) mode, with Alice transmitting information signal while Bob emits AN. We then jointly design beamforming and AN vectors to degrade the reception signal quality at Eve, based on the signal-to-interference-plus-noise ratio (SINR) of each node. To tackle the power minimization problem, we propose an iterative algorithm that includes an additional constraint to ensure adherence to specified quality-of-service (QoS) metrics. Additionally, we decompose the robust optimization problem of the two schemes into two sub-problems, with one that can be solved using generalized Rayleigh quotient methods and the other that can be addressed through semi-definite programming (SDP). Finally, our simulation results confirm the viability of the proposed approach and demonstrate the effectiveness of the protection zone for NFC systems operating with CSI.

## 1. Introduction

### 1.1. Background

Physical layer security (PLS) is a pivotal field of research in wireless communications, encompassing a range of security techniques that leverage the inherent randomness of wireless channels to protect exchanged information [1,2]. With the broadcast nature of wireless signal propagation and the rapid technological advancements available to potential eavesdroppers, ensuring secure communications presents a challenging task. This endeavor is rooted in information theory, introduced by Claude Shannon in 1949 [3], providing a framework to ascertain the capacity limits of communication systems. By designing a system where the communication capacity between a transmitter (Tx) and the intended receiver (Rx) exceeds that with potential eavesdroppers, a measurable secrecy capacity can be achieved. This forms the basis of PLS, with its foundation laid by Wyner’s introduction of the wiretap channel model in 1975 [4].

With the growing demand for high-quality wireless communications, there will be a shift towards ultra-large-scale antenna arrays and high-frequency communications. This trend makes electromagnetic (EM) wave signals propagating more likely in the near-field region rather than the usual far-field region. The boundary between the near-field and far-field regions is determined by the Rayleigh distance, which is proportional to the square of the array aperture and is inversely proportional to the signal carrier wavelength [5]. Specially, when an Rx is located in the far-field region, the EM field propagation is approximately modeled by planar waves. However, in the near-field region, near-field propagation becomes dominant and the EM field propagation needs to be accurately modeled by spherical waves. Unlike the plane wave model that only contains angular information for beamforming, the spherical wave propagation model includes both the distance and angular information of the Rx, enabling the Tx to focus a beam on a specific point in near-field communications (NFCs). Therefore, NFCs can leverage distance and angle to achieve more precise signal enhancement or interference cancellation. As a result, new opportunities in wireless communications, such as accurate interference management, enhanced multiplexing gains, and simultaneous angle and distance estimation, have garnered significant attention in recent studies [6,7]. Additionally, secure communication and resource allocation are applicable in various fields [8,9,10], making research on near-field communication particularly urgent.

The characteristics of NFCs provide an effective solution to the challenges of PLS. The beamfocusing in NFCs allows the energy of the artificial noise (AN) signal to concentrate at the location of illegal users rather than just towards their direction, effectively improving the jamming efficiency, reducing the interference of AN to the intended Rx and thus improving the system secrecy capacity. More specifically, even if both legal and illegal users are located in the same direction, they can still be distinguished in the distance domain, resulting in lower information leakage rate. Therefore, by optimizing the beamfocusing vectors at the Tx, the potential of NFCs for enhancing PLS can be fully exploited, which is the motivation of this work.

### 1.2. Related Works

The growing interest in the MIMO (multiple-input multiple-output) technique enables the exploitation of spatial degrees of freedom (DoF) to enhance the secrecy capacity of wireless communication systems. Researchers have delved into the MIMO wiretap channel and developed techniques to enhance communication secrecy [11,12,13], which considers the case of multiple antennas at all nodes and is termed as the multiple-input multiple-output multiple-antenna eavesdropper (MIMOME) channel. Khisti et al. [12] developed a genie-aided upper bound for the MIMO secrecy capacity for which Gaussian inputs are optimal. When the eavesdropper’s instantaneous channel state information (CSI) is known at the Tx, it was shown that an asymptotically optimal scheme had to apply a transmit precoder based upon the generalized singular value decomposition (GSVD) of the pencil $\left(H_{B},H_{E}\right)$, where $H_{B}$ and $H_{E}$ are the channel matrices for the Tx-to-Rx and Tx-to-eavesdropper links, respectively, which decomposes the system into parallel channels and leads to a closed-form secrecy rate expression. Additionally, Negi and Goel improved the communication secrecy rate using AN in systems where the number of transmit antennas exceeds that of the receiving antennas [11], i.e., jamming. Specifically, based on the CSI provided by the legitimate Rx, the Tx places AN in the null space of the channel matrix associated with the legitimate Rx. Depending on the channel matrix between the Tx and the eavesdropper, some component of AN will be projected into its range space, thereby degrading the signal-to-interference-plus-noise ratio (SINR) at the eavesdropper. Subsequently, several practical issues related to the MIMO and multiple-input single-output (MISO) wiretap channel [12,13] were considered, and the optimal power allocation methods for the AN strategy were developed in [14,15]. Jamming as a kind of active PLS scheme is beneficial as it can provide a guaranteed secrecy rate, regardless of the eavesdropper’s channel quality, which is difficult to control or even obtain in practice [16].

Furthermore, several works [13,17,18,19,20] have investigated optimal beamforming designs under the assumption that the eavesdropper CSI can be perfectly obtained at the Tx. This assumption has been relaxed in other works to scenarios where the Tx has only partial or no information about the eavesdropper CSI. If statistical information regarding the eavesdroppers’ channel is unavailable, Swindlehurst et al. [21] suggested an approach where just enough power is allocated to meet a target performance criterion (SINR or rate) of the Rx, and all the remaining power is used for broadcasting AN, since the secrecy rate cannot be computed at the Tx. The effects of imperfect CSI at the Tx upon achieving the secrecy rate were examined in [22,23]. When the potential CSI of an eavesdropper is not perfectly known at the Tx, i.e., the CSI of the eavesdropper suffers a norm-bounded mismatch, or even when the Tx has no access to the passive eavesdropper’s CSI, Derrick et al. [24] considered a multi-user multiple-input single-output (MUMISO) downlink system with simultaneous wireless information and power transfer and aimed to jointly design the transmit beamforming, AN, and energy signals. Considering stochastic models, in [25,26] studied the achievable secrecy rate maximization and the outage probability minimization of MISO eavesdropping channels, respectively.

It is important to note that the PLS schemes mentioned above are based on the traditional far-field channel model. As the communication carrier frequency increases and the antenna aperture grows, the traditional far-field model becomes inaccurate and near-field propagation needs to be considered for PLS. Several initial studies utilizing the distance dimension in the spherical-wave channel model to enhance the security of wireless communications have been proposed [27,28,29,30,31,32]. In [28,29], analog beam focusing techniques were investigated to enhance near-field PLS, with a focus on the hardware challenges of XL-arrays. Specifically, in [29], an analog directional modulation precoding algorithm was proposed to secure transmissions at both the angular and distance scales for a MISO system. Further research has highlighted the benefits of beam focusing for near-field secure transmission. For instance, in [30] demonstrated that beam focusing can significantly improve jamming rejection and secrecy performance. Additionally, in [31], a secure transmission scheme was developed to combat cooperative eavesdropping in a spherical-wave channel model, focusing on distance-domain security. Finally, in [32] introduced a near-field secure transmission framework using classical hybrid beamforming, emphasizing that the security of near-field communications is largely determined by the relative distance between the eavesdropper and the legitimate user. Yet, these studies assume that the base station (BS) has obtained the perfect CSI of all nodes. Therefore, the dedicated secrecy beamfocusing strategy for the MIMOME channel with imperfect CSI still needs further investigation.

### 1.3. Main Contributions

In this paper, we consider the near-field PLS problem with the partial CSI of the eavesdropper with multiple antennas at all nodes. Our approach specifically utilizes CSI for precise beamforming by incorporating both the angular and distance information. This enhances the focus of the signal on the legitimate receiver while reducing the risk of eavesdropping. Additionally, we strategically allocate the AN using the CSI, ensuring it disrupts the eavesdropper without interfering with legitimate communication. The system’s capability to manage imperfect CSI is enhanced, making it more resilient under real-world conditions. These innovations represent significant advancements in the PLS of NFC systems, distinguishing this approach from the traditional methods. Our main contributions are as follows:

- We consider two schemes for near-field secure communication in a unified scenario. Generally, we refer to the Tx, Rx, and eavesdropper as Alice, Bob, and Eve, respectively, all of whom have multiple antennas. In Scheme I, which we call the Tx-AN scheme, Alice simultaneously transmits signals and emits AN to hinder Eve, who is positioned close to Alice, from eavesdropping on the legitimate information. In Scheme II, known as the Rx-AN scheme, Alice transmits signals while Bob operates in full-duplex mode, enabling him to both receive signals and transmit AN simultaneously. This approach aims to prevent Eve, who is close to Bob, from capturing any information. We formulate the robust design of beamforming and AN vectors as a power minimization problem, taking into account the QoS constraint on Bob, the security constraint on Eve, and the total transmission power requirements in the presence of imperfect CSI of the eavesdropping channel.
- To solve the non-convex optimization problem of the joint transmit beamforming, receive beamforming, and AN beamforming design, we decompose the original robust optimization problem into two sub-problems and propose an iterative strategy based on the alternating optimization principle, as follows: (1) When the beamforming and AN vectors at Alice are given, updating the receiving beamforming vector at the Bob simplifies to a generalized Rayleigh quotient problem, which can be solved in a closed form; (2) When the receiving beamforming vector at the receiving end is given, the joint design of the transmit beamforming and AN vectors can be reformulated as a semi-definite programming (SDP) problem and can be solved efficiently by an off-the-shelf convex optimization approach.
- To illustrate the advantages of NFC for PLS more intuitively, we conducted simulations examining the relationship between average transmit power versus several factors, i.e., the number of antennas, the position of Eve. This allowed us to utilize near-field beams for enhancing jamming efficiency. Simulation results indicate that we should select the node closer to Eve for transmitting AN to counter eavesdropping. This strategy not only improves the efficiency of AN transmission but also increases the likelihood of creating a near-field propagation condition that is favorable for jamming.

The rest of this paper is organized as follows: In Section 2, we introduce the NFC system model and channel model. Section 3 presents the problem formulation. In Section 4, we present the proposed algorithms that can give a suboptimal solution of the corresponding optimization problem. Then, we present the numerical results with discussions in Section 5. Finally, we conclude the paper with Section 6.

Annotations: Lower-case letters are used to represent scalars, while vectors and matrices are denoted by lower-case and upper-case boldface letters, respectively. The set of complex numbers is denoted by C. *ℜ* extracts the real part of a complex number. For vector x, $x_{j}$ denotes the *j*-th element of x, and $\mathrm{diag}\left(x\right)$ denotes a diagonal matrix with its diagonal entries given by x. For matrix A, $A^{T}$, $A^{H}$, ${∥A∥}_{F}$, $A^{-1}$, $\mathrm{rank}\left(A\right)$, and $\mathrm{tr}\left(A\right)$ denote the matrix transpose, conjugate transpose, Frobenius norm, inverse, rank, and trace of A, respectively. ${\left[A\right]}_{ij}$ extracts the (i,j)-th element of matrix A. CN(μ,Σ) denotes a circularly symmetric complex Gaussian random vector distribution with mean μ and covariance matrix Σ.

## 2. System Model

**Figure 1:**
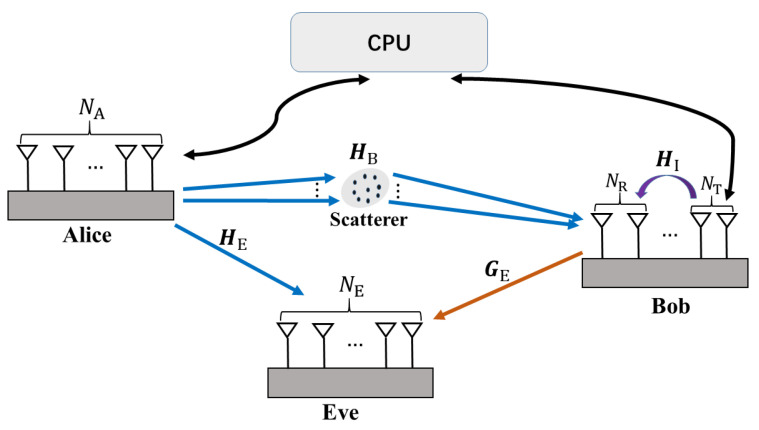
The near-field secure wireless communication system.

In this section, we first introduce the system model for the two proposed schemes in a downlink near-field secure communication scenario, as illustrated in Figure 1. Then, we present the channel models and CSI error model for the proposed schemes.

### 2.1. System Model for the Proposed Two Schemes

We consider a massive MIMO secure downlink transmission system which consists of a transmitter (Alice), a legitimate receiver (Bob), and an eavesdropper (Eve). Alice, Bob, and Eve are equipped with $N_{A}$, $N_{B}=N_{T}+N_{R}$ (where $N_{T}$ is the number of transmit antennas at Bob, and $N_{R}$ is the number of receive antennas) and $N_{E}$ antennas, respectively, i.e., MIMOME channel. Bob is able to decode the information transmitted by Alice, while Eve’s goal is to illicitly eavesdrop on Alice’s information. We assume that the coordinates of the Alice, Bob, and Eve are $q_{A}=\left(0,0,0\right)$, $q_{B}=\left(x_{B},y_{B},0\right)$, and $q_{E}=\left(x_{E},y_{E},0\right)$, respectively. Without a loss of generality, we assume that all nodes have the same height, which is set to 0 for convenience in the subsequent steps. Thus, the $n_{A}$-th antenna of Alice is located at $\left(x_{n_{A}},y_{n_{A}},0\right)$. Similarly, the coordinates of the $n_{B}$-th antenna of Bob and the $n_{E}$-th antenna of Eve are $\left(x_{n_{B}},y_{n_{B}},0\right)$ and $\left(x_{n_{E}},y_{n_{E}},0\right)$, respectively.

#### 2.1.1. Tx-AN

The Tx-AN scheme allows Alice to transmit both information signal and AN to Bob and Eve, where AN is introduced to protect the information signal. Its important to note that $N_{R}=0$, i.e., $N_{B}=N_{T}$ in this scheme. Denoted by $x=ws+v\in C^{N_{A}\times 1}$ is the transmitted signal from Alice to Bob, in which *s* is the information symbol that follows CN(0,1), $w\in C^{N_{A}\times 1}$ is the precoding vector for the information signal at Alice, and $v\in C^{N_{A}\times 1}$ is the AN vector generated by Alice to combat Eve. The downlink received signals at Bob and Eve are given by the following:

(1) $$\begin{array}{cc} y_{B} & =r_{B}^{H}\left(H_{B}x+n_{B}\right)\in C^{1\times 1},\mathrm{and} \end{array}$$

(2) $$\begin{array}{cc} y_{E} & =r_{E}^{H}\left(H_{E}x+n_{E}\right)\in C^{1\times 1}, \end{array}$$

where $r_{B}\in C^{N_{B}\times 1}$ and $r_{E}\in C^{N_{E}\times 1}$ are the unit-norm receiving beamforming vectors at Bob and Eve, respectively. $H_{B}\in C^{N_{B}\times N_{A}}$ is the channel matrix between Alice and Bob, which can be perfectly obtained by both of them. $H_{E}\in C^{N_{E}\times N_{A}}$ is the channel matrix between Alice and Eve. Since Eve will not transmit a training sequence to Alice and may only receive pilot signals from Alice, we assume that Eve perfectly knows its CSI $H_{E}$, while Alice only has partial information about $H_{E}$, denoted as ${\hat{H}}_{E}$. $n_{B}$ and $n_{E}$ are the additive white Gaussian noises (AWGN) with zero mean and variance $\sigma_{B}^{2}$ and $\sigma_{E}^{2}$, respectively.

The achievable rate of Bob is given by the following:

(3) $$\begin{array}{c} R_{B}=\log_{2}\left(1+\mathrm{SINR}\right)=\log_{2}\left(\frac{1+r_{B}^{H}H_{B}ww^{H}H_{B}^{H}r_{B}}{r_{B}^{H}H_{B}vv^{H}H_{B}^{H}r_{B}+\sigma_{B}^{2}}\right), \end{array}$$

where ${\mathrm{SINR}}_{B}$ is the received signal-to-interference plus noise ratio (SINR) at Bob. On the other hand, the leakage information rate of Eve is given by the following:

(4) $$\begin{array}{c} R_{E}=\log_{2}\left(1+{\mathrm{SINR}}_{E}\right)=\log_{2}\left(\frac{r_{E}^{H}H_{E}ww^{H}H_{E}^{H}r_{E}}{r_{E}^{H}H_{E}vv^{H}H_{E}^{H}r_{E}+\sigma_{E}^{2}}\right), \end{array}$$

where ${\mathrm{SINR}}_{E}$ is the received SINR at Eve.

#### 2.1.2. Rx-AN

In the Rx-AN scheme, we consider that Bob is operating in the full-duplex mode and is capable of simultaneously receiving the information transmitted by Alice and emitting AN to interfere with Eve. The numbers of antennas for Alice, Bob, and Eve remain the same as before, but Bob operates in the full-duplex mode with $N_{R}$ receiving antennas and $N_{T}$ transmitting antennas; $N_{B}=N_{T}+N_{R}$. $x=ws\in C^{N_{A}\times 1}$ is the transmitted signal from Alice to Bob, and $v\in C^{N_{T}\times 1}$ is the AN vector generated by Bob. Then, the downlink received signals at Bob and Eve are given by the following:

(5) $$\begin{array}{cc} y_{B} & =r_{B}^{H}\left(H_{B}ws+\sqrt{\rho }H_{I}v+n_{B}\right)\in C^{1\times 1},\mathrm{and} \end{array}$$

(6) $$\begin{array}{cc} y_{E} & =r_{E}^{H}\left(H_{E}ws+G_{E}v+n_{E}\right)\in C^{1\times 1}, \end{array}$$

where $H_{B}\in C^{N_{R}\times N_{A}}$, and $H_{E}\in C^{N_{E}\times N_{A}}$ are the channel matrices of the Alice-to-Bob and Alice-to-Eve links, respectively. $G_{E}\in C^{N_{E}\times N_{T}}$ is the channel matrix from Bob to Eve. $H_{I}\in C^{N_{R}\times N_{T}}$ is the self-interference channel from transmitting antennas to receiving antennas at Bob and ρ is the residual factor. When ρ is 0, it means that self-interference has been completely eliminated [33].

The achievable rate of Bob is given by the following:

(7) $$\begin{array}{c} R_{B}=\log_{2}\left(1+{\mathrm{SINR}}_{B}\right)=\log_{2}\left(\frac{r_{B}^{H}H_{B}ww^{H}H_{B}^{H}r_{B}}{\rho r_{B}^{H}H_{I}vv^{H}H_{I}^{H}r_{B}+\sigma_{B}^{2}}\right), \end{array}$$

The leakage information rate of Eve is given by following:

(8) $$\begin{array}{c} R_{E}=\log_{2}\left(1+{\mathrm{SINR}}_{E}\right)=\log_{2}\left(\frac{r_{E}^{H}H_{E}ww^{H}H_{E}^{H}r_{E}}{r_{E}^{H}G_{E}vv^{H}G_{E}^{H}r_{E}+\sigma_{E}^{2}}\right). \end{array}$$

### 2.2. Channel Models

In this section, we first introduce the channel models corresponding to the two proposed schemes and then present the CSI availability.

Following the traditional Saleh–Valenzuela (SV) channel model, the Alice-to-Bob channel can be given by a far-field channel model in the literature [34], as follows:

(9) $$H_{B}=\sqrt{\frac{N_{A}N_{B}}{L}}\sum_{l=1}^{L}\gamma a_{N_{B}}\left(\theta_{B}^{l}\right)a_{N_{A}}^{H}\left(\theta_{A}^{l}\right),$$

where $\gamma =\frac{\lambda^{2}}{{\left(4\pi d_{\mathrm{AB}}\right)}^{2}}e^{-j\frac{2\pi }{\lambda }d_{\mathrm{AB}}}$ is the path coefficient, λ is wavelength of the carrier frequency signal, *L* is the total number of paths, and $d_{\mathrm{AB}}$ is the distance between Alice and Bob. Moreover, $a_{N_{A}}\left(\theta_{A}^{l}\right)\in C^{N_{A}\times 1}$ and $a_{N_{B}}\left(\theta_{B}^{l}\right)\in C^{N_{B}\times 1}$ denote the array response vectors at the Alice and Bob, respectively, which are given by the following:

(10) $$\begin{array}{cc} a_{N_{A}}\left(\theta_{A}^{l}\right) & ={\left[e^{-j\frac{2\pi }{\lambda }\frac{\left(1-N_{A}\right)}{2}d\;\cos\;\theta_{A}^{l}},\cdots ,e^{-j\frac{2\pi }{\lambda }\frac{\left(N_{A}-1\right)}{2}d\;\cos\;\theta_{A}^{l}}\right]}^{T},\mathrm{and} \end{array}$$

(11) $$\begin{array}{cc} a_{N_{B}}\left(\theta_{B}^{l}\right) & ={\left[e^{-j\frac{2\pi }{\lambda }\frac{\left(1-N_{B}\right)}{2}d\;\cos\;\theta_{B}^{l}},\cdots ,e^{-j\frac{2\pi }{\lambda }\frac{\left(N_{B}-1\right)}{2}d\;\cos\;\theta_{B}^{l}}\right]}^{T}, \end{array}$$

where $\theta_{A}^{l}$ is the azimuth angle-of-departure (AoD) from Alice to Bob, while $\theta_{B}^{l}$ is the azimuth angle-of-arrival (AoA) at Bob. For the Alice-to-Eve link, as Eve might be placed closer to Alice to improve its eavesdropping efficiency, $H_{E}\in C^{N_{E}\times N_{A}}$ needs to be accurately modeled according to the near-field channel model [32,35,36].

(12) $$H_{E}=\alpha_{n_{E}n_{A}}b_{N_{E}}\left(d_{n_{E}n_{A}}\right)a_{N_{A}}^{H}\left(\psi_{A}\right),$$

where $\alpha_{n_{E}n_{A}}=\frac{\lambda^{2}}{{\left(4\pi d_{n_{E}n_{A}}\right)}^{2}}$ is the associated path coefficient, and $b_{N_{E}}\left(d_{n_{E}n_{A}}\right)\in C^{N_{E}\times 1}$ is the near-field steering vector, which is given by the following:

(13) $$b_{N_{E}}\left(d_{n_{E}n_{A}}\right)={\left[e^{-j\frac{2\pi }{\lambda }d_{11}},\cdots ,e^{-j\frac{2\pi }{\lambda }d_{1N_{E}}}\right]}^{T},$$

where the distance between the $n_{A}$-th antenna at Alice and the $n_{E}$-th element at Eve is given by the following:

(14) $$d_{n_{E}n_{A}}=\sqrt{{\left(x_{n_{E}}-x_{n_{A}}\right)}^{2}+{\left(y_{n_{E}}-y_{n_{A}}\right)}^{2}}.$$

The definition of $a_{N_{A}}\left(\psi_{A}\right)\in C^{N_{A}\times 1}$ is similar to that of $a_{N_{A}}\left(\theta_{A}^{l}\right)$ above, and $\psi_{A}$ is the azimuth AoD from Alice to Eve. Moreover, Eve might also approach Bob to improve the leakage information rate as the channels for the Alice-to-Bob and Alice-to-Eve links become highly correlated in this case. Therefore, the Bob-to-Eve channel $G_{E}\in C^{N_{E}\times N_{T}}$ also needs to be defined following the near-field channel model, which is given by the following equation:

(15) $$\begin{array}{c} G_{E}=\alpha_{n_{E}n_{T}}b_{N_{E}}\left(d_{n_{E}n_{T}}\right)a_{N_{T}}^{H}\left(\psi_{B}\right), \end{array}$$

where $\alpha_{n_{E}n_{T}}=\frac{\lambda^{2}}{{\left(4\pi d_{n_{E}n_{T}}\right)}^{2}}$ is the associated path coefficient, and $b_{N_{E}}\left(d_{n_{E}n_{T}}\right)\in C^{N_{E}\times 1}$ is the near-field steering vector similar to (13), where $d_{n_{E}n_{T}}$ is the distance between the $n_{T}$-th antenna at Bob and the $n_{E}$-th element at Eve. The definition of $a_{N_{T}}\left(\psi_{B}\right)\in C^{N_{T}\times 1}$ is similar to (10) and $\psi_{B}$ is the azimuth AoD from Bob to Eve. Note that when Eve is located far from Alice or Bob, i.e., in the far-field region, $H_{E}$ and $G_{E}$ naturally degenerate to the far-field channel, respectively.

Additionally, the self-interference channel $H_{I}$ typically modeled as a near-field channel [37,38], as follows:

(16) $$\begin{array}{c} {\left[H_{I}\right]}_{n_{R}n_{T}}=\alpha_{n_{R}n_{T}}e^{-j\frac{2\pi }{\lambda }d_{n_{R}n_{T}}}, \end{array}$$

where $\alpha_{n_{R}n_{T}}=\frac{\lambda^{2}}{{\left(4\pi d_{n_{R}n_{T}}\right)}^{2}}$ is the associated path coefficient, and $d_{n_{R}n_{T}}$ is the distance between the $n_{T}$-th transmit antennas and teh $n_{R}$-th receive antennas at Bob.

We outline the availability of CSI for each node in the two schemes in Table 1. The legitimate nodes upload their acquired CSI to a central processing unit (CPU) for the joint design. In the Tx-AN scheme, Alice can transmit her desired signal along with AN simultaneously, while Bob exclusively receives the signal. This results in a lower requirement for CSI. Conversely, in the Rx-AN scheme, Alice only transmits the desired signal, while Bob operates in the full-duplex mode, meaning he both receives the signal and transmits AN at the same time, which results in a higher requirement for CSI.

### 2.3. CSI Error Models

Since Eve is assumed to have the capability and resources to fully estimate her own channel parameters, we assume that Eve perfectly knows $H_{E}$ and $G_{E}$. And Alice and Bob only have partial information of $H_{E}$ and $G_{E}$, following some wiretap channel estimation methods [39,40]. In particular, to model the wiretap channel uncertainty, the channel of Alice-to-Eve and Bob-to-Eve are given by the following equations:

(17) $$\begin{array}{cc} H_{E} & ={\hat{H}}_{E}+\Delta H_{E},\mathrm{and} \end{array}$$

(18) $$\begin{array}{cc} G_{E} & ={\hat{G}}_{E}+\Delta G_{E}, \end{array}$$

(19) $$\begin{array}{cc} \Omega_{H} & ≜\{\Delta H_{E}\in C^{N_{E}\times N_{A}}:\;∥\Delta H_{E}{∥}_{F}^{2}\leq \varepsilon_{H}^{2}\},\mathrm{and} \end{array}$$

(20) $$\begin{array}{cc} \Omega_{G} & ≜\{\Delta G_{E}\in C^{N_{E}\times N_{T}}:\;∥\Delta G_{E}{∥}_{F}^{2}\leq \varepsilon_{G}^{2}\}, \end{array}$$

where ${\hat{H}}_{E}$ and ${\hat{G}}_{E}$ are the channel estimates of the Alice-to-Eve and Bob-to-Eve links, respectively, and $\Delta H_{E}$ and $\Delta G_{E}$ represent the corresponding channel estimation errors (CEEs). Moreover, $\Delta H_{E}$ and $\Delta G_{E}$ are bounded within the sets $\Omega_{H}$ and $\Omega_{G}$, respectively, as defined in (19) and (20). These sets characterize the CSI uncertainties, where $\varepsilon_{H},\varepsilon_{G}>0$ represent the bounds of the CEEs.

It is important to note that the availability of partial CSI creates a trade-off between security performance and system complexity. While perfect CSI allows for optimal beamforming and the allocation of AN to maximize security, partial CSI can reduce the accuracy of signal targeting and AN allocation. This may lead to lower secrecy rates and an increased risk of eavesdropping. To mitigate these issues, robust algorithms are necessary, which in turn increases computational complexity and resource usage. Despite these challenges, systems that utilize partial CSI are generally more practical and resilient under real-world conditions. Striking a balance between robustness to CSI uncertainty and system efficiency is essential to ensure both security and practicality in future communication networks.

### 2.4. The Design of $r_{\;E}$

Since the CSI of Eve is unavailable, it is not possible to design her beamforming vector adaptively. As a result, we can only predefine her beamforming vector in advance based on assumptions or specific scenarios to account for her potential behavior in the system analysis.

For the Tx-AN scheme, due to the CPU only knowing partial channel information from Alice to Eve and Eve being unable to obtain w and v from Alice, we can assume that the worst-case maximum ratio combining (MRC) $r_{E}$ is adopted at Eve. We fix $r_{E}$ as the left eigenvector corresponding to the maximum eigenvalue of ${\hat{H}}_{E}$, i.e., ${\hat{H}}_{E}=U_{E}^{I}\Sigma_{E}^{I}{D_{E}^{I}}^{H}$, then $r_{E}={U_{E}^{I}}_{\left(:,1\right)}$.

For the Rx-AN scheme, when designing $r_{E}$, Eve does not know w transmitted by Alice and v transmitted by Bob. Additionally, Alice and Bob only know ${\hat{H}}_{E}$ and ${\hat{G}}_{E}$. Therefore, we assume $ww^{H}$ and $vv^{H}$ are both identity matrices. Then, $r_{E}$ can be designed as follows:

(21) $$\begin{array}{cc} r_{E}=\arg\max_{∥r_{E}∥=1}{\mathrm{SINR}}_{E} & =\arg\max_{∥r_{E}∥=1}\frac{r_{E}^{H}{\hat{H}}_{E}{\hat{H}}_{E}^{H}r_{E}}{r_{E}^{H}\left({\hat{G}}_{E}{\hat{G}}_{E}^{H}+\sigma_{E}^{2}I_{N_{E}}\right)r_{E}},\\ \; & =P({\hat{H}}_{E}{\hat{H}}_{E}^{H},{\hat{G}}_{E}{\hat{G}}_{E}^{H}+\sigma_{E}^{2}I_{N_{E}}), \end{array}$$

where P(·) denotes the operator that returns principle generalized eigenvector based on the two input matrices [41], and the optimal solution can be given by the following equation:

(22) $$\begin{array}{c} {\hat{H}}_{E}{\hat{H}}_{E}^{H}r_{E}=\mu_{\max}\cdot \left({\hat{G}}_{E}{\hat{G}}_{E}^{H}+\sigma_{E}^{2}I_{N_{E}}\right)r_{E}\Rightarrow Fr_{E}=\mu_{\max}r_{E}, \end{array}$$

where $F={\left({\hat{G}}_{E}{\hat{G}}_{E}^{H}+\sigma_{E}^{2}I_{N_{E}}\right)}^{-1}{\hat{H}}_{E}{\hat{H}}_{E}^{H}$. We then perform eigenvalue decomposition on F, i.e., $F=U_{E}^{II}\Sigma_{E}^{II}{D_{E}^{II}}^{H}$, where  $r_{E}=U_{E}^{II}\left(:,1\right)$ is the left eigenvector corresponding to the largest eigenvalue $\mu_{\max}$.

**Remark** **1.**  *The system design utilizes NFCs combined with massive MIMO arrays to enhance PLS. This is achieved through precise beamforming and effective interference management, utilizing both angular and distance information to tackle the challenges posed by imperfect CSI. While this method significantly increases the secrecy capacity, there are opportunities for future enhancements. These could include adaptive beamforming systems that provide real-time countermeasures against eavesdropping strategies and the incorporation of advanced anti-jamming and anti-spoofing techniques for improved security. Furthermore, machine learning could be employed to predict the behavior of potential eavesdroppers and dynamically adjust beamforming strategies, thereby offering a more resilient and intelligent defense against evolving eavesdropping tactics.*

## 3. Problem Formulation

In this section, we present the problem formulation. For the Tx-AN scheme, we aim to design the optimal beamfoming vector w, the AN vector v, and the receiving beamforming vector $r_{B}$ to minimize the total transmit power which is formulated as the following optimization problem:

(23) $$\begin{array}{cc} \left(P1\right)\;\min_{w,v,r_{B}} & {\left||w|\right|}_{2}^{2}+{\left||v|\right|}_{2}^{2}\\ \;s.t.\;\;\;C1: & {\mathrm{SINR}}_{B}\geq \Gamma_{\mathrm{Req}},\\ \;\;\;\;C2: & \max_{\Delta H_{E}\in \Omega_{H}}{\mathrm{SINR}}_{E}\leq \Gamma_{\mathrm{Tol}},\\ \;\;\;\;C3: & {\left||w|\right|}_{2}^{2}+{\left||v|\right|}_{2}^{2}\leq P_{\max}. \end{array}$$

In C1, $\Gamma_{\mathrm{Req}}$ denotes the minimum SINR of Bob required for information decoding. Constraint C2 is imposed such that for a given CSI uncertainty set $\Omega_{H}$, the maximum received SINR at Eve is less than the maximum tolerable received SINR $\Gamma_{\mathrm{Tol}}$. In practice, we have $\Gamma_{\mathrm{Req}}\gg \Gamma_{\mathrm{Tol}}>0$, to ensure secure communication. C3 limits the total transmit power at Alice with $P_{\max}$ as the power budget.

Similar to the Tx-AN scheme, for the Rx-AN scheme, our goal is also to jointly optimize the beamforming vector w, the AN vector v, and the receiving beamforming vector $r_{B}$. The optimization problem for the Rx-AN scheme is given by the following:

(24) $$\begin{array}{cc} \left(P2\right)\;\min_{w,v,r_{B}} & {\left||w|\right|}_{2}^{2}+{\left||v|\right|}_{2}^{2}\\ \;s.t.\;\;\;C1: & {\mathrm{SINR}}_{B}\geq \Gamma_{\mathrm{Req}},\\ \;\;\;\;C2: & \max_{\Delta H_{E}\in \Omega_{H},\Delta G_{E}\in \Omega_{G}}{\mathrm{SINR}}_{E}\leq \Gamma_{\mathrm{Tol}},\\ \;\;\;\;C3: & {\left||w|\right|}_{2}^{2}\leq P_{A}{,||v||}_{2}^{2}\leq P_{B} \end{array}$$

Constraint C3 imposes separate power limits on the transmit beamforming vector w and the AN vector v, ensuring that their respective powers do not exceed $P_{A}$ and $P_{B}$. However, to enable a fair comparison between the Tx-AN scheme and the Rx-AN scheme, we introduce a total power constraint $P_{\max}=P_{A}+P_{B}$, where $P_{A}$ and $P_{B}$ denote the power allocated to w and v, respectively. The power constraints in the Tx-AN and Rx-AN schemes are vital for efficiently allocating transmit power between the beamforming vector and the AN vector. This balance maximizes signal quality for the Bob while creating interference for the Eve. In both schemes, the total transmit power, $P_{\max}$, is divided into $P_{A}$ and $P_{B}$, which represent the power allocated to the beamforming vector and the AN vector, respectively. As our scenario is uniform, this setup is designed to ensure fairness, enabling us to identify which scheme is more effective at resisting eavesdropping in the following simulations.

These two optimization problems both have three variables that need to be updated, including $w,r_{B},\;\mathrm{and}\;v$, while $r_{E}$ is prefixed. We will update the two sets of variables separately based on an alternating optimization framework, which will be presented in Section 4.

## 4. Alternating Optimization Algorithm for the Power Minimization Problem with Imperfect CSI

In this section, we develop an efficient algorithm which optimizes {w,v} and $r_{B}$ in an alternating manner. In particular, the original problem with respect to $r_{B}$ becomes a generalized Rayleigh quotient problem [41] when {w,v} are given, while the optimization of {w,v} with the given $\left\{r_{B}\right\}$ can be transformed into a convex problem by the S-procedure [42].

### 4.1. Alternating Optimization Algorithm for Tx-AN Scheme

Given w and v, the receiving beamforming vector $r_{B}$ at Bob is given by maximizing its received SINR, as follows:

(25) $$\begin{array}{cc} r_{B}=\arg\max_{∥r_{B}∥=1}{\mathrm{SINR}}_{B} & =\frac{r_{B}^{H}H_{B}ww^{H}H_{B}^{H}r_{B}}{r_{B}^{H}H_{B}vv^{H}H_{B}^{H}r_{B}+\sigma_{B}^{2}},\\ \; & =\arg\max_{∥r_{B}∥=1}\frac{r_{B}^{H}H_{B}WH_{B}^{H}r_{B}}{r_{B}^{H}\left(H_{B}VH_{B}^{H}+\sigma_{B}^{2}I_{N_{B}}\right)r_{B}},\\ \; & =P(H_{B}WH_{B}^{H},H_{B}VH_{B}^{H}+\sigma_{B}^{2}I_{N_{B}}), \end{array}$$

where $W=ww^{H}$, and $V=vv^{H}$. The solution to the optimization problem (25) can be directly given as follows:

(26) $$\begin{array}{c} {\left(H_{B}VH_{B}^{H}+\sigma_{B}^{2}I_{N_{B}}\right)}^{-1}H_{B}WH_{B}^{H}r_{B}=\lambda_{\max}\cdot r_{B}\Rightarrow Cr_{B}=\lambda_{\max}r_{B}, \end{array}$$

where $C={\left(H_{B}VH_{B}^{H}+\sigma_{B}^{2}I_{N_{B}}\right)}^{-1}H_{B}WH_{B}^{H}$. To perform eigenvalue decomposition on C, i.e., $C=U_{B}^{I}\Sigma_{B}^{I}{D_{B}^{I}}^{H}$, then $r_{B}=U_{B}^{I}\left(:,1\right)$ is the left eigenvector corresponding to the maximum eigenvalue of $\lambda_{\max}$.

When $r_{B}$ is given, the optimization problem in (23) can be reformulated as follows:

(27) $$\begin{array}{cc} \;\;\min_{W,V} & \mathrm{Tr}(W+V)\\ s.t.\;C1: & {\mathrm{SINR}}_{B}\geq \Gamma_{\mathrm{Req}},\\ \;\;\;\;C2: & \max_{\Delta H_{E}\in \Omega_{H}}{\mathrm{SINR}}_{E}\leq \Gamma_{\mathrm{Tol}},\\ \;\;\;\;C3: & \mathrm{Tr}\left(W+V\right)\leq P_{\max},\\ \;\;\;\;C4: & W⪰0,V⪰0,\\ \;\;\;\;C5: & \mathrm{rank}(W)=1,\mathrm{rank}(V)=1. \end{array}$$

where constraints C4 and C5 in (27) are imposed to guarantee that $W=ww^{H}$ and $V=vv^{H}$ hold well after optimizing W and V. The main obstacle to solve the problem in (27) is C2, which includes semi-infinite constraints. Fortunately, C2 can be transformed to a linear matrix inequality (LMI) using the following lemma.

**Lemma** **1**  (General S-procedure [43])**.** *Define the quadratic functions of the variable $x\in C^{n\times 1}$ as follows:*

(28) $$\begin{array}{c} f_{m}\left(x\right)=x^{H}A_{m}x+2ℜ\left\{b_{m}^{H}x\right\}+c_{m},m=0,\cdots ,M, \end{array}$$

*where $A_{m}\in H^{n}$, $b_{m}\in C^{n\times 1}$, and $c_{m}\in R$. Then, the implication ${\left\{f_{m}\left(x\right)\geq 0\right\}}_{m=1}^{M}$⇒$f_{0}\left(x\right)\geq 0$ holds if, and only if, there exist $\delta_{m}\geq 0,\forall m$ such that the following is achieved:*

(29) $$\left[\begin{array}{cc} A_{0} & b_{0}\\ b_{0}^{H} & c_{0} \end{array}\right]-\sum_{m=1}^{M}\delta_{m}\left[\begin{array}{cc} A_{m} & b_{m}\\ b_{m}^{H} & c_{m} \end{array}\right]⪰0.$$

Substituting $h_{E}={\hat{h}}_{E}+\Delta h_{E}$ into constraint C2, the implication is as follows:

(30) $$\begin{array}{cc} \; & \Delta h_{E}^{H}\Delta h_{E}\leq \varepsilon_{H}^{2}\\ \; & \Rightarrow \Delta h_{E}^{H}\left(W-\Gamma_{\mathrm{Tol}}V\right)\Delta h_{E}+2ℜ\left\{{\hat{h}}_{E}^{H}\left(W-\Gamma_{\mathrm{Tol}}V\right)\Delta h_{E}\right\}+{\hat{h}}_{E}^{H}\left(W-\Gamma_{\mathrm{Tol}}V\right){\hat{h}}_{E}-\Gamma_{\mathrm{Tol}}\sigma_{E}^{2}\leq 0 \end{array}$$

which holds if, and only if, there exists a $\delta_{E}\geq 0$, such that the following LMI constraint holds:

(31) $$\begin{array}{cc} \; & C2:S_{C2}^{1}\left(W,V,\delta_{E}\right)\\ \; & =\left[\begin{array}{cc} \delta_{E}I_{N_{A}}+\Gamma_{\mathrm{Tol}}V & \Gamma_{\mathrm{Tol}}V{\hat{h}}_{E}\\ \Gamma_{\mathrm{Tol}}{\hat{h}}_{E}^{H}V & \Gamma_{\mathrm{Tol}}\sigma_{E}^{2}+\Gamma_{\mathrm{Tol}}{\hat{h}}_{E}^{H}V{\hat{h}}_{E}-\delta_{E}\varepsilon_{H}^{2} \end{array}\right]-T^{H}WT⪰0, \end{array}$$

where $T=[I_{N_{A}}\;{\hat{h}}_{E}]$.

Substituting (31) into (27), we obtain the following optimization problem:

(32) $$\begin{array}{cc} \;\;\min_{W,V,\delta_{E}} & \mathrm{Tr}(W+V)\\ s.t.\;C1: & \mathrm{Tr}\left(h_{B}h_{B}^{H}\left(W-\Gamma_{\mathrm{Req}}V\right)\right)-\Gamma_{\mathrm{Req}}\sigma_{B}^{2}\geq 0,\\ \;\;\;\;C2: & S_{C2}^{1}\left(W,V,\delta_{E}\right)⪰0,\\ \;\;\;\;C3: & \mathrm{Tr}\left(W+V\right)\leq P_{\max},\\ \;\;\;\;C4: & W⪰0,V⪰0,\\ \;\;\;\;C5: & \mathrm{rank}(W)=1,\mathrm{rank}(V)=1,\\ \;\;\;\;C6: & \delta_{E}\geq 0. \end{array}$$

Now, the only remaining obstacle to solve (24) is constraint C5. By removing this constraint, the problem becomes a convex semi-definite programming (SDP) issue. This can be efficiently solved using numerical solvers like SeDuMi and SDPT3 [44,45]. From the basic principles of optimization theory, if the obtained solution W and V for the relaxed problem admits a rank-one matrix, then it is the optimal solution of the original problem in (P1). Then, the optimal w and v can be obtained by performing eigenvalue decomposition on W and V.

For the Tx-AN scheme, the algorithm is summarized as follows: This process aims to minimize the total transmit power while satisfying constraints such as SINR requirements and power limits. When $W^{\left(n-1\right)}$ and $V^{\left(n-1\right)}$ are given, the objective function of the original problem becomes constant, i.e., $\mathrm{Tr}(W^{\left(n-1\right)}+V^{\left(n-1\right)})=C$, where *C* is a constant. We only need to satisfy the constraints for $r_{B}^{\left(n-1\right)}$, which forms a generalized Rayleigh quotient problem. On the other hand, when $r_{B}^{\left(n-1\right)}$ is given, the problem with respect to W and V can be transformed into an SDP problem, which will reduce the total transmit power, i.e., $\mathrm{Tr}\left(W^{\left(n\right)}+V^{\left(n\right)}\right)\leq \mathrm{Tr}\left(W^{\left(n-1\right)}+V^{\left(n-1\right)}\right)$. In summary, the convergence of the algorithm is guaranteed, and each sub-problem can achieve an optimal solution.

Step 1 involves solving a matrix inverse and an eigen decomposition problem of dimension $N_{B}$, hence the computational complexity of this step is $O(N_{B}^{3})$. Step 2 is an SDP problem with variables of dimension $N_{A}\times N_{A}$, resulting in a computational complexity of $O(N_{A}^{3.5})$. Once it converges, we need to perform an SVD on $W^{\left(n\right)}$, with a computational complexity of $O(N_{A}^{3})$. Hence, the overall computational complexity of Algorithm 1 is $O(T\left(N_{A}^{3.5}+N_{B}^{3}\right)+N_{A}^{3})$, where *T* is the number of iterations.
**Algorithm 1:** AO-based algorithm for solving (P1)
1: Input: $H_{B}$, ${\hat{H}}_{E}$, $P_{\max}$, $\Gamma_{\mathrm{Req}}$, $\Gamma_{\mathrm{Tol}}$ and threshold ϵ.
2: Output: w, $r_{B}$, $r_{E}$ and V
3: Initialization: Let the iteration index to be n=0 and $n_{\max}=100$. Initialize $W^{\left(0\right)}$, $V^{\left(0\right)}$ to be $N_{A}\times N_{A}$ positive semi-definite matrices with $w^{\left(0\right)}$ and $v^{\left(0\right)}$ are the right singular vector and basis vector in the null space of $H_{B}$, respectively. $r_{E}=U_{E}^{I}\left(:,1\right)$.
4: repeat
Step 1. Given $W^{\left(n-1\right)}$ and $V^{\left(n-1\right)}$, update $r_{B}^{\left(n-1\right)}$:$$r_{B}^{\left(n-1\right)}=P\left(H_{B}W^{\left(n-1\right)}H_{B}^{H},H_{B}V^{\left(n-1\right)}H_{B}^{H}+\sigma_{B}^{2}I\right).$$
Step 2. Given $r_{B}^{\left(n-1\right)}$ and $r_{E}$, update $W^{\left(n\right)}$ and $V^{\left(n\right)}$: Solve the Problem in (27).

5: until $|P^{\left(n\right)}-P^{\left(n-1\right)}|<\epsilon$, the rank-one solution w can be obtained by singular value decomposition (SVD) of $W^{\left(n\right)}$. Otherwise, n←(n+1) and repeat Step 1 and Step 2.


The alternating optimization algorithm effectively scales with the number of antennas. However, as the number of antennas increases, the computational complexity and memory requirements grow significantly, potentially becoming a bottleneck for real-time processing. To address scalability in large MIMO systems, techniques like parallel computing and approximation algorithms can be employed. Additionally, the algorithm’s frequency-independent nature ensures consistent performance across different frequency ranges, making it versatile for various applications. In summary, while the AO algorithm is effective for medium-sized systems, advanced methods are needed to maintain scalability and efficiency in large MIMO systems.

### 4.2. Alternating Optimization Algorithm for Rx-AN Scheme

Similar to the algorithm for the Tx-AN scheme, when W and V are given, the receiving beamforming vector $r_{B}$ at Bob is obtained by maximizing its received SINR, as follows:

(33) $$\begin{array}{cc} r_{B}=\arg\max_{∥r_{B}∥=1}{\mathrm{SINR}}_{B} & =\arg\max_{∥r_{B}∥=1}\frac{r_{B}^{H}H_{B}WH_{B}^{H}r_{B}}{r_{B}^{H}\left(\rho H_{I}VH_{I}^{H}+\sigma_{B}^{2}I_{N_{E}}\right)r_{B}},\\ \; & =P(H_{B}WH_{B}^{H},\rho H_{I}VH_{I}^{H}+\sigma_{B}^{2}I_{N_{E}}), \end{array}$$

Then, $r_{B}$ can be obtained by the principle generalized eigenvector, which is given by the following:

(34) $$\begin{array}{c} H_{B}WH_{B}^{H}r_{B}=\gamma_{\max}\cdot \left(\rho H_{I}VH_{I}^{H}+\sigma_{B}^{2}I_{N_{E}}\right)r_{B}\Rightarrow Dr_{B}=\gamma_{\max}r_{B}, \end{array}$$

where $D={\left(\rho H_{I}VH_{I}^{H}+\sigma_{B}^{2}I_{N_{E}}\right)}^{-1}H_{B}WH_{B}^{H}$ and eigenvalue decomposition is performed on D, i.e., $D=U_{B}^{II}\Sigma_{B}^{II}{D_{B}^{II}}^{H}$, and $r_{B}=U_{B}^{II}\left(:,1\right)$ is the left eigenvector corresponding to the largest eigenvalue $\gamma_{\max}$.

When $r_{B}$ is given, the optimization problem in (22) can be reformulated as follows:

(35) $$\begin{array}{cc} \;\;\min_{W,V} & \mathrm{Tr}(W)+\mathrm{Tr}(V)\\ s.t.\;C1: & {\mathrm{SINR}}_{B}\geq \Gamma_{\mathrm{Req}},\\ \;\;\;\;C2: & \max_{\Delta H_{E}\in \Omega_{H},\Delta G_{E}\in \Omega_{G}}{\mathrm{SINR}}_{E}\leq \Gamma_{\mathrm{Tol}},\\ \;\;\;\;C3: & \mathrm{Tr}\left(W\right)\leq P_{A},\mathrm{Tr}\left(V\right)\leq P_{B},\\ \;\;\;\;C4: & W⪰0,V⪰0,\\ \;\;\;\;C5: & \mathrm{rank}(W)=1,\mathrm{rank}(V)=1. \end{array}$$

Substituting $h_{E}={\hat{h}}_{E}+\Delta h_{E}$ and $g_{E}={\hat{g}}_{E}+\Delta g_{E}$ into constraint C2, the implication is as follows:

(36) $$\begin{array}{c} \Delta h_{E}^{H}\Delta h_{E}\leq \varepsilon_{H}^{2}\Rightarrow x^{H}\left[\begin{array}{cc} I_{N_{A}} & 0\\ 0 & 0 \end{array}\right]x-\varepsilon_{H}^{2}\leq 0, \end{array}$$

(37) $$\begin{array}{c} \Delta g_{E}^{H}\Delta g_{E}\leq \varepsilon_{G}^{2}\Rightarrow x^{H}\left[\begin{array}{cc} 0 & 0\\ 0 & I_{N_{T}} \end{array}\right]x-\varepsilon_{G}^{2}\leq 0, \end{array}$$

where $x^{H}={\left[\Delta h_{E}^{H}\;\Delta g_{E}^{H}\right]}^{H}$. After some manipulation, we obtain the following:

(38) $$x^{H}\left[\begin{array}{cc} W & 0\\ 0 & -\Gamma_{\mathrm{Tol}}V \end{array}\right]x+2ℜ\left\{{\left[\begin{array}{c} W{\hat{h}}_{E}\\ -\Gamma_{\mathrm{Tol}}V{\hat{g}}_{E} \end{array}\right]}^{H}x\right\}+c\leq 0,$$

where $c={\hat{h}}_{E}^{H}W{\hat{h}}_{E}-\Gamma_{\mathrm{Tol}}{\hat{g}}_{E}^{H}V{\hat{g}}_{E}-\Gamma_{\mathrm{Tol}}\sigma_{E}^{2}$. Then, there must exist $\delta_{H},\delta_{G}\geq 0$, such that the following LMI constraint holds:

(39) $$\begin{array}{cc} \; & C2:S_{C2}^{2}\left(W,V,\delta_{H},\delta_{G}\right)\\ \; & =\left[\begin{array}{cc} \left[\begin{array}{cc} W & 0\\ 0 & -\Gamma_{\mathrm{Tol}}V \end{array}\right] & \left[\begin{array}{c} W{\hat{h}}_{E}\\ -\Gamma_{\mathrm{Tol}}V{\hat{g}}_{E} \end{array}\right]\\ {\left[\begin{array}{c} W{\hat{h}}_{E}\\ -\Gamma_{\mathrm{Tol}}V{\hat{g}}_{E} \end{array}\right]}^{H} & c \end{array}\right]-\delta_{H}\left[\begin{array}{cc} \left[\begin{array}{cc} -I_{N_{A}} & 0\\ 0 & 0 \end{array}\right] & 0\\ 0 & \varepsilon_{H}^{2} \end{array}\right]-\delta_{G}\left[\begin{array}{cc} \left[\begin{array}{cc} 0 & 0\\ 0 & -I_{N_{T}} \end{array}\right] & 0\\ 0 & \varepsilon_{G}^{2} \end{array}\right]⪰0. \end{array}$$

Substituting (39) into (35), we obtain the following optimization problem:

(40) $$\begin{array}{cc} \;\;\min_{W,V,\delta_{H},\delta_{G}} & \mathrm{Tr}(W)+\mathrm{Tr}(V)\\ s.t.\;C1: & \mathrm{Tr}\left(h_{B}h_{B}^{H}W\right)-\rho \Gamma_{\mathrm{Req}}\mathrm{Tr}\left(h_{I}h_{I}^{H}V\right)-\Gamma_{\mathrm{Req}}\sigma_{B}^{2}\geq 0,\\ \;\;\;\;C2: & S_{C2}^{2}\left(W,V,\delta_{H},\delta_{G}\right)⪰0,\\ \;\;\;\;C3: & \mathrm{Tr}\left(W\right)\leq P_{A},\mathrm{Tr}\left(V\right)\leq P_{B},\\ \;\;\;\;C4: & W⪰0,V⪰0,\\ \;\;\;\;C5: & \mathrm{rank}(W)=1,\mathrm{rank}(V)=1,\\ \;\;\;\;C6: & \delta_{H},\delta_{G}\geq 0. \end{array}$$

The algorithm for the Rx-AN scheme closely resembles that of the Tx-AN scheme. Therefore, further explanations and discussions regarding its convergence and computational complexity will not be elaborated upon here.

The paper proposes two secure near-field communication schemes, Tx-AN and Rx-AN, for a uniform scenario. It details the methodology for designing beamforming vectors and implementing AN strategies. The work introduces a robust optimization framework that utilizes alternating optimization to address the power minimization problem under conditions of imperfect CSI. This approach effectively breaks down the non-convex problem into manageable sub-problems. The iterative algorithm is explained step-by-step, ensuring transparency and reproducibility while addressing the challenges associated with imperfect CSI, all while maintaining signal quality and security. Additionally, the paper incorporates mathematical formulations, including the generalized Rayleigh quotient problem and SDP, to optimize beamforming and AN allocation. This results in a rigorous and well-structured method for solving design challenges in secure communication.>

**Remark** **2.**  *The study addresses critical challenges in NFC by proposing two secure communication schemes that operate under imperfect CSI. It develops robust strategies for beamforming and AN allocation to enhance PLS, which is essential for future high-quality wireless systems. Using near-field propagation models, particularly spherical waves, the research optimizes beamforming by taking into account both angular and distance factors. An iterative algorithm for power minimization is introduced to tackle the non-convex nature of the problem, effectively addressing real-world issues such as imperfect CSI. Overall, the paper offers an effective solution to enhance PLS in NFC systems, focusing on practical issues and their applicability in real-world scenarios.*

## 5. Simulations

In this section, we evaluate the performance of the proposed resource allocation schemes through simulations. We consider a 3D Cartesian coordinate system where Alice and Bob are positioned at (0,0,0) m and (0,100,0) m, respectively. The location of Eve may vary depending on the channel conditions, placing her in the near-field region of either Alice or Bob. For ease of analysis, when Eve is close to Alice, her position is (0,20,0) m, and when Eve is close to Bob, her position is (0,90,0) m. Alice is equipped with $N_{A}=128$ transmit antennas, which correspond to a Rayleigh distance of $r_{\mathrm{Ray}}^{A}=80.7$ m. Bob has $N_{B}=64$ receive antennas, with a Rayleigh distance of $r_{\mathrm{Ray}}^{B}=20.5$ m. Eve has $N_{E}=32$ antennas. The carrier frequency is 30 GHz. Unless specified otherwise, the system parameters are described as follows: $\sigma_{B}^{2}=\sigma_{E}^{2}=-110$ dBm, $P_{\max}=40$ dBm, $\Gamma_{\mathrm{Req}}=10$ dB, $\Gamma_{\mathrm{Tol}}=0$ dB, $\epsilon_{H}=\epsilon_{G}=0.1$, and $\epsilon =10^{-3}$. Choosing $\Gamma_{\mathrm{Req}}=10$ dB ensures reliable communication with sufficient SINR for high-order modulation schemes, while $\Gamma_{\mathrm{Tol}}=0$ dB enhances security by preventing decoding by Eve. This approach is commonly utilized in practical systems like LTE and 5G, balancing communication reliability and security. The numerical results are obtained by averaging over 50 channel realizations. Specifically, we consider the following benchmark schemes for performance comparison:

- Baseline 1: Maximum ratio transmission (MRT) is adopted for the beamforming matrix W, i.e., $W=\sqrt{\frac{1}{\mathrm{Tr}(H_{B}H_{B}^{H})}}H_{B}^{H}$, and the AN covariance matrix V is chosen to lie in the null space of $H_{B}$, such that the AN does not degrade the channel quality of the Bob.
- Baseline 2: The beamforming matrix W also adopts the MRT strategy. However, the AN covariance matrix V is chosen to lie in the null space of $H_{I}$, which helps to minimize the effects of self-interference as much as possible. The self-interference factor is set as ρ=0.1.

In Baseline 2, the AN vector is selected in the null space of the self-interference channel matrix $H_{I}$. This design ensures that the noise has minimal impact on Bob’s signal while effectively jamming Eve. This strategy protects the communication by preventing Eve from detecting the noise while ensuring clear signal reception for Bob, thereby achieving a balance between security and performance.

### 5.1. Convergence Behavior

**Figure 2:**
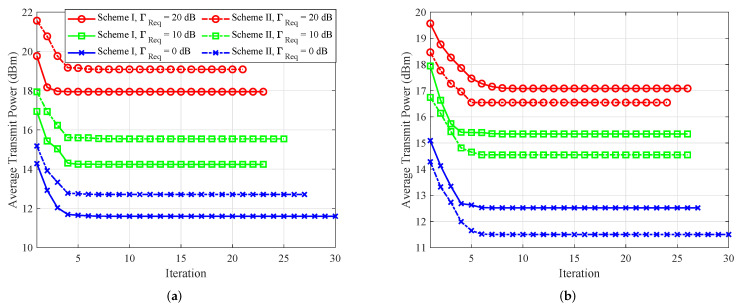
Convergence behavior of the proposed algorithm for both schemes. (**a**) When Eve is within the near-field region of Alice. (**b**) When Eve is within the near-field region of Bob.

In Figure 2, we separately verify the convergence of the proposed AO algorithms for both schemes. Comparing Figure 2a,b, it can be seen that the AN should be emitted by the party that Eve is closer to, which leads to more effective jamming.

### 5.2. Average Total Power Versus Minimum Required SINR

**Figure 3:**
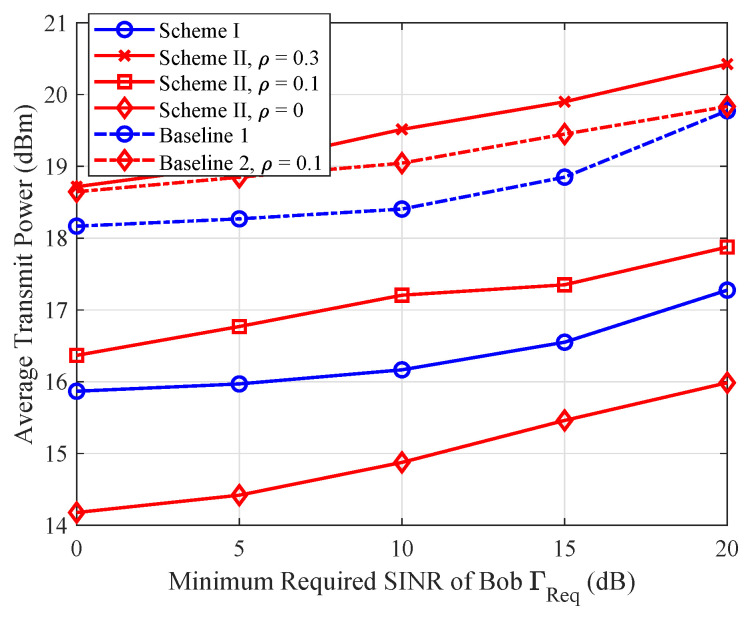
The average transmit power versus minimum required SINR $\Gamma_{\mathrm{Req}}$.

In Figure 3, we present the average transmit power versus the minimum required SINR $\Gamma_{\mathrm{Req}}$ of Bob, while limiting the maximum tolerable SINR at Eve as $\Gamma_{\mathrm{Tol}}=0$ dB. From Figure 3, we observe that more transmit power is required to satisfy the increased SINR of Bob. By comparing our proposed methods with the baseline schemes, it is seen that the power consumption of the proposed scheme is lower than the baseline scheme. The reason is that the baseline scheme only exploits the channel information of Bob, thus their power consumption stays at a high level. However, as ρ increases to 0.1 and above, we observe that the power required for Scheme II exceeds that of Scheme I. This is because Bob requires more power to counteract the AN propagated through self-interference channel.

### 5.3. Average Total Power Versus $N_{\;A}$ and $N_{E}$

**Figure 4:**
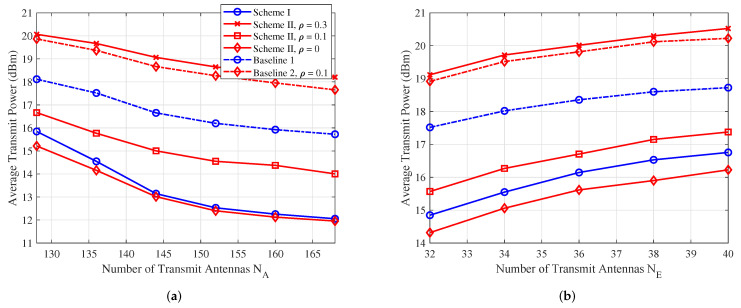
(**a**) The average transmit power versus number of Alice’s antennas $N_{A}$. (**b**) The average transmit power versus number of Eve’s antennas $N_{E}$.

In Figure 4, we examine the average transmit power versus the number of Alice’s antennas $N_{A}$ and the number of Eve’s antennas $N_{E}$, respectively. The minimum required SINR $\Gamma_{\mathrm{Req}}=10$ dB, and the maximum tolerable SINR at Eve is $\Gamma_{\mathrm{Tol}}=0$ dB. In Figure 4a, it is clear that the average transmit power decreases as the number of transmit antennas at Alice increases. Conversely, Figure 4b shows that the average transmit power increases as the number of receive antennas at Eve grows. Moreover, comparing the two proposed algorithms with Baselines 1 and 2, it is evident that the proposed algorithms significantly save transmit power, leading to more energy-efficient transmission.

### 5.4. Average Total Power Versus the Position of Eve

**Figure 5:**
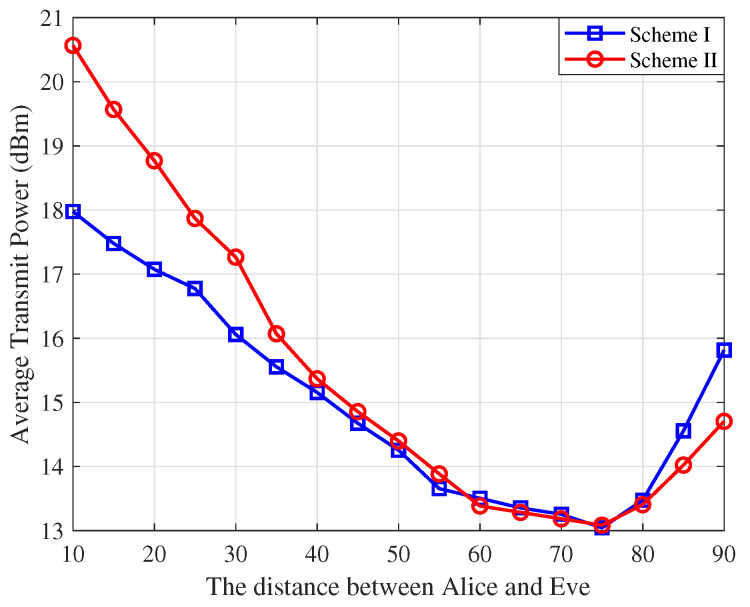
The average transmit power versus the distance between Alice and Eve.

In Figure 5, we present the average transmit power versus the distance between Alice and Eve for both schemes. The results reveal three distinct regions with $r_{\mathrm{Ray}}^{A}=80.7$ m and $r_{\mathrm{Ray}}^{B}=20.5$ m. In the middle region (distances between 30 m and 70 m), the average power consumption is minimized for both schemes, as the eavesdropping capability of Eve weakens due to the increasing distance from both Alice and Bob, resulting in reduced power allocation for AN. When Eve is close to Bob (distances above 70 m), the correlation between $H_{E}$ and $H_{B}$ becomes significant, requiring Alice to increase the AN power to ensure secrecy in Sscheme I. On the other hand, when Eve is close to Alice (distances below 30 m), Scheme II requires Bob to allocate more AN power, as Eve is close to Alice which enhances her ability to intercept the information signal. Scheme I is more effective for short-range communication (up to 30–40 m), while Scheme II excels in long-range communication, compensating better for fading and interference. These results demonstrate that the power allocation strategies in both schemes are highly dependent on the position of Eve. This adaptability ensures efficient resource usage and enhanced security.

### 5.5. Protection Zone for the Proposed Two Schemes

To demonstrate the advantage of near-field secure communication with the imperfect CSI of Eve, inspired by [46,47], we conducted power heat map calculations on the plane where Alice, Bob, and Eve are located. Below are the desired signal power $P_{d}={\left|HWW^{H}H\right|}_{F}$ and the interference-plus-noise power $P_{i}={\left|HVV^{H}H+\sigma_{E}^{2}I\right|}_{F}$ corresponding to Schemes I and II, respectively. $P_{d}$ represents the desired signal power, which measures the strength of the received signal at the intended receiver (Bob). On the other hand, $P_{i}$ denotes the interference-plus-noise power, which includes both interference and noise at Eve’s receiver. The protection zone refers to the spatial area in which AN is used to prevent Eve from decoding the signal while ensuring that Bob remains unaffected. Near-field effects play a role in shaping the interference pattern within this zone, making it sensitive to Eve’s position and the transmission configuration, particularly when her receiver is in close proximity.

**Figure 6:**
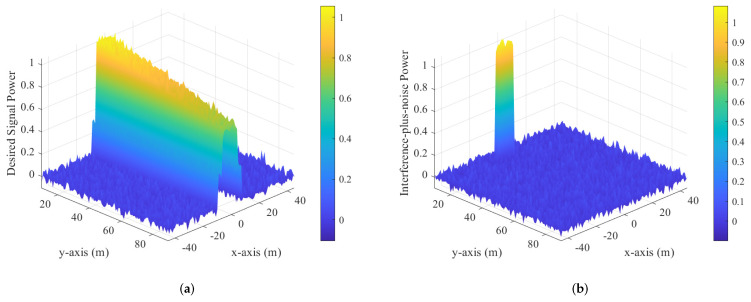
Normalized power heat maps for Scheme I. (**a**) Desired signal power. (**b**) Interference-plus-noise power.

In Figure 6a, the power of the desired signal varies according to Bob’s position. The highest power is concentrated nearest to Alice, highlighting the strength of the direct link between Alice and Bob. As the distance between them increases, the signal power gradually decreases, illustrating the effects of path loss and reduced signal strength associated with greater separation. In Figure 6b, the distribution of interference-plus-noise power is depicted, demonstrating how Alice utilizes AN to create a protection zone around Eve. In this zone, interference levels are elevated, which effectively hinders Eve’s ability to eavesdrop. By comparing Figure 6a,b, it becomes clear that Alice’s allocation of AN successfully shields the area surrounding Eve, forming a protective barrier that diminishes her capacity to intercept the communication.

**Figure 7:**
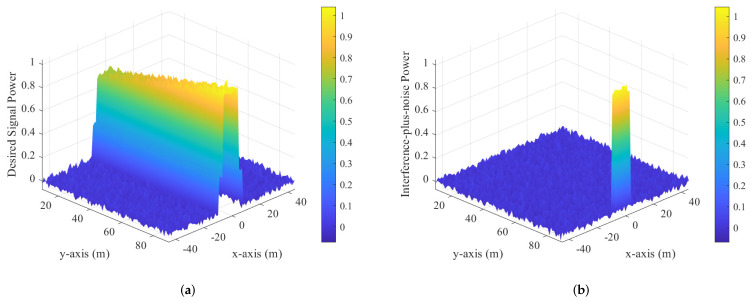
Normalized power heat maps for scheme II. (**a**) Desired signal power. (**b**) Interference-plus-noise power.

Figure 7 presents the normalized power heat maps for Scheme II; Figure 7a shows the desired signal power and Figure 7b shows the interference-plus-noise power on a 2D plane where Alice, Bob, and Eve are located. In Figure 7a, the desired signal power is highest near Bob, demonstrating the effectiveness of Scheme II in focusing the transmit power towards the intended receiver. As the distance from Bob increases, the signal power gradually decreases due to path loss, showcasing a localized and spatially concentrated communication channel. In Figure 7b, the interference-plus-noise power heat map highlights the role of Bob’s full-duplex AN generation. The interference power is elevated in regions near Eve, effectively creating a protection zone that degrades Eve’s ability to intercept the communication. In summary, these heat maps illustrate the capability to maintain high desired signal power near Bob while creating a significant interference barrier around Eve. Additionally, a more effective jamming strategy can be chosen based on the position of Eve.

## 6. Conclusions

This paper proposes teh following two schemes for secure downlink near-field communication: the Tx-AN scheme and the Rx-AN scheme. We employ alternating optimization algorithms to address challenges related to resource allocation. By fixing the transmit beamforming vector and the covariance matrix of the AN, we optimized the receive beamforming for both Bob and Eve. Similarly, when the receive beamforming vectors were held constant, optimizing the transmit beamforming and the covariance matrix of AN transformed the issue into an SDP problem. Our findings indicate that near-field communication can effectively protect legitimate user communications from eavesdropping by degrading Eve’s channel quality, even when there is imperfect CSI. Simulation results further demonstrate that near-field beam focusing enhances jamming efficiency. By selecting a node closer to Eve to emit AN, we can achieve secure communication while also promoting energy efficiency and reducing emissions. Future research could investigate models that consider Eve to have the imperfect CSI of Alice or Bob. This could involve incorporating statistical channel estimation errors or acknowledging bounded uncertainty in Eve’s acquisition of CSI. Additionally, adaptive algorithms could be developed to adjust security levels based on the estimated quality of the CSI that Eve can acquire, enabling a more effective balance between security and resource efficiency.

## Figures and Tables

**Table 1:** CSI availability for the proposed two schemes.

Scheme	Alice	Bob	Eve	CPU
Tx-AN	$H_{B}$, ${\hat{H}}_{E}$	$H_{B}$	$H_{E}$	$H_{B}$, ${\hat{H}}_{E}$
Rx-AN	$H_{B}$, ${\hat{H}}_{E}$	$H_{B}$, $H_{I}$, ${\hat{G}}_{E}$	$H_{E}$, $G_{E}$	$H_{B}$, $H_{I}$, ${\hat{H}}_{E}$, ${\hat{G}}_{E}$

## Data Availability

Data are contained within the article.

## Abbreviations

The following abbreviations are used in this manuscript:

AN	Artificial noise
AoA	Angle-of-arrival
AoD	Angle-of-departure
AWGN	Additive white Gaussian noises
BS	Base station
CEEs	Channel estimation errors
CPU	Central processing unit
CSI	Channel estimation information
EM	Electromagnetic
FD	Full-duplex
GSVD	Generalized singular value decomposition
MIMO	Multiple-input multiple-output
MIMOME	Multiple-input multiple-output multiple-antenna eavesdropper
MISO	Multiple-input single-output
MUMISO	Multi-user multiple-input single-output
NFCs	Near-field communications
PLS	Physical layer security
QoS	Quality-of-service
Rx	Receiver
SDP	Semi-definite programming
SINR	Signal-to-interference-plus-noise ratio
SV	Saleh-Valenzuela
SVD	Singular value decomposition
Tx	Transmitter
